# Specific Condis Crystal-like Mesophase of Poly(butylene succinate-*co*-butylene adipate)

**DOI:** 10.1021/acssuschemeng.4c03285

**Published:** 2024-06-11

**Authors:** Khadar Duale, Henryk Janeczek, Marcin Godzierz, Ákos György Juhász, Joanna Rydz

**Affiliations:** † Centre of Polymer and Carbon Materials, Polish Academy of Sciences, M. Curie-Skłodowska 34, 41-819 Zabrze, Poland; ‡ Laboratory of Nanochemistry Department of Biophysics and Radiation Biology Semmelweis University, Nagyvárad tér 4, Budapest H-1089, Hungary

**Keywords:** poly(butylene succinate-*co*-butylene adipate), PBSA, condis crystal, liquid crystal, conformationally distorted crystal

## Abstract

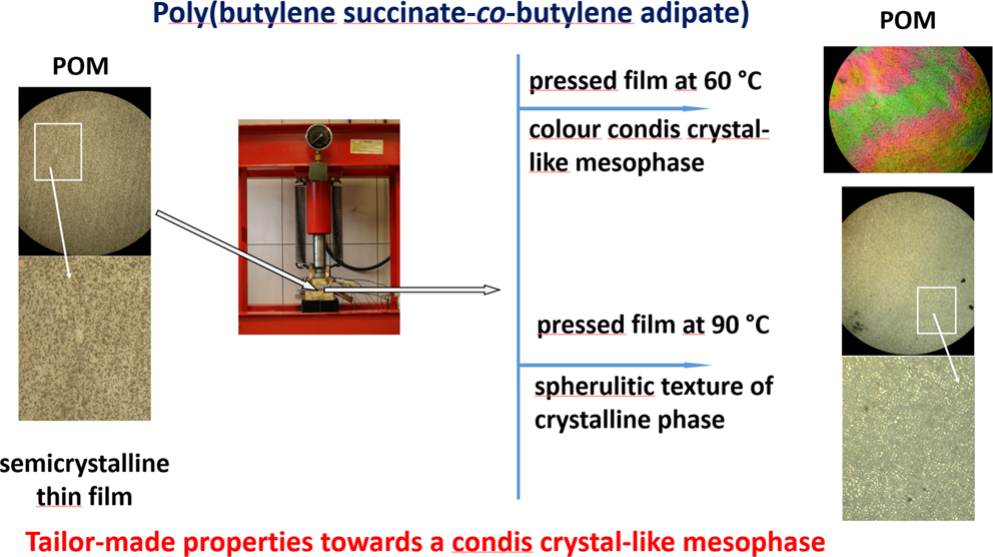

Understanding the properties of polymers, such as their crystallinity, is crucial for their material performance and predicting their behavior during and after use, especially in the case of environmentally friendly (bio)degradable polymers, enabling optimized design. In this work, for the first time, a pressure-induced condis crystal-like mesophase of poly(butylene succinate-*co*-butylene adipate) (PBSA) is presented. The phase behavior of pressed films obtained from commercial PBSA with 25% butylene adipate units is investigated at various processing temperatures from room temperature to 100 °C, pressed at a pressure of the press jaws and at 2–5 t for 1–5 min. The characterization and quantification evaluation of the condis crystal-like mesophase of pressed PBSA formed at temperatures above the glass transition is investigated by X-ray diffraction, polarized optical microscopy (POM), and differential scanning calorimetry (DSC) methods. Our results demonstrate that pressed PBSA films at 60 °C show a condis crystal-like mesophase, characterized by the presence of reflections at wide angles, birefringence by POM, as well as a higher melting point (endotherm) by DSC. The resulting oriented mesomorphic green polymer can, in a sustainable manner, expand further technological applications of (bio)degradable polymers, especially in the medical field, and open up opportunities for further research that could provide such polymers with tailored persistence and degradation, thus changing the shelf life.

## Abstract 2

For the first time, a pressure-induced condis crystal-like mesophase of poly(butylene succinate-*co*-butylene adipate) is presented, which may provide polymers with improved durability and tailored degradation in a sustainable manner.

## Introduction

Understanding the morphology of polymer structures under various processing conditions is crucial for controlling their thermal and mechanical properties, but this is also important with regard to their susceptibility to degradation both under usage and after having been discarded. Polymers display intricate morphological structures compared to other materials, emphasizing the need to understand the diversity of their molecular composition, molar mass, supramolecular structures, molecular orientation, and finally, phase morphology to fully elucidate their degradation behavior. This also helps to unlock their full potential and adapt production processes to achieve the desired properties, enabling the creation of sustainable polymers with specific tailor-made properties, for various applications. Therefore, a thorough understanding of morphology remains essential to optimize polymer performance and functionality.^1^

Knowledge of the thermodynamic properties, particularly the crystallization mode of polymers, is an essential part of their material characterization as this provides information about their properties, as well as their behavior during and after use. A large number of molecules with mesophase properties have been identified, but there are undoubtedly many more compounds and macromolecules that are mesomorphic other than those identified so far. Mesophases are of interest to researchers from different disciplines owing to their increasingly widespread practical application and their occurrence in many different systems, such as biological systems.^2−7^ The types of mesophase that have been identified so far include liquid crystals (LCs), conformationally disordered crystals (condis), and plastic crystals.^8−12^ LCs can be created in single- and multicomponent systems. However, they can only occur in substances, whose molecules have the right structure, i.e., a highly anisotropic geometric molecular shape or amphiphilicity.^13,14^ In the case of polymers, the presence of rigid parts, such as those in aromatic groups, is required.^1^ However, as ever, there are exceptions. In one of our previous studies on (bio)degradable polymers, in particular, polylactide (PLA), the characteristics of PLA mesophases were reported. The polymer films were exposed to pressure and temperature, whereby a nematic mesophase with varying textures was formed at temperatures below or above the glass-transition temperature (*T*_g_) of PLA but below its melting point (melting temperature, *T*_m_).^15^

The other mesophase found in some linear macromolecules with flexible chains includes conformationally disordered crystal-type structures called condis crystals. It was Wunderlich, who suggested that this new type of mesophase for this system should be recognized and proposed to call it a conformationally disordered state or condis crystal.^2^ Semicrystalline polymers with linear chains can usually form condis crystals. Semicrystalline polymers are interspersed with highly ordered crystalline regions and other regions, in which the chains are disordered or amorphous. The crystalline regions exhibit well-defined polymer chain conformations, while the amorphous regions contain disordered conformations. The long-range orientational order of both nematic LC and condis crystal mesophase is observed as the presence of birefringence in cross-polarized light.^8^ In contrast to LCs, condis crystals do not contain rigid rod- or disc-like mesogen groups dispersed in a matrix of another polymer.^16^ In addition, the liquid-like and positional perturbations, which are characteristic of LCs, are not present in condis crystals. Likewise, there are no orientation disturbances and rotational movements of the molecules in the plastic crystal phase.^17^ Conformational disorder within crystalline regions can also be observed in semicrystalline polymers, impacting their properties and behavior. For example, polyethylene (PE) has been observed to form a condis crystal mesophase at high temperature and pressure.^18^ The best results are achieved if two mesophase polymers, such as PE and poly(bis(2,2,2-trifluoroethoxy)phosphazene), are used in the same binary mixture.^16^ Similar observations were reported for PLA processed above the *T*_g_ at 74, 85, and 120 °C, as it displayed a condis crystal-like mesophase.^1^

(Bio)degradable polymers have been perceived as green, environmentally friendly and potential alternatives to conventional plastics in a wide range of applications and industries.^3,4^ The aliphatic polyester poly(butylene succinate-*co*-butylene adipate) (PBSA) is a biodegradable and biocompatible thermoplastic polymer of great interest. PBSA copolyesters of various compositions were synthesized either by polycondensation from 1,4-butanediol and diacids or by transesterification from 1,4-butanediol and succinic and adipic acid dimethyl esters.^19^ Therefore, this copolymer results in a material that combines the properties of both monomeric units, butylene succinate and butylene adipate, making PBSA versatile and therefore suitable for many different applications in a variety of fields, including packaging, textiles, agriculture, and medicine.^20−23^ PBSA is known to biodegrade more slowly than polyhydroxyalkanoates (PHAs) but much faster than poly(butylene succinate) (PBS). It also has lower crystallinity compared to PBS and is therefore suggested for film applications.^24^ PBSA is reported to crystallize in the same crystal lattice as PBS but the butylene adipate group in the PBSA copolymer reduces crystallinity as well as the size of the crystal lamellae.^25^

In general, mesomorphic polymers have been found to provide several advantages due to their oriented nature, including increased tensile strength and impact toughness, reduced degradation rate, longer shelf life, lower gas permeability, and improved thermal conductivity with low electrical conductivity.^26^ These properties make mesomorphic polymers highly suitable for a variety of applications. However, few (bio)degradable polymers have been successfully commercialized, mainly because mesogenic moieties need to be present within the polymer for it to naturally exhibit mesomorphic properties.^1^ The formation of conformationally disordered crystal behaviors for PBSA is reported here, for the first time. The obtained results confirm that the pressed PBSA films have outstanding ability to self-assemble into a mesophase under the influence of mechanical pressure, time, and heat. Understanding conformational distortions in polymer chains is important for the design of green polymers with specific properties, such as flexibility or thermal stability. The PBSA films were characterized by differential scanning calorimetry (DSC), polarized optical microscope (POM), and X-ray diffraction (XRD).

## Experimental Section

### Materials

PBSA pellets (PBE 001) with 25% of butylene adipate units (as determined by proton nuclear magnetic resonance spectroscopy) and with mass-average molar mass *M*_w_ = 180,000 g mol^–1^, molar mass dispersity *Đ*_M_ = 2.6 (measured by gel permeation chromatography), *T*_g_ = −43.4 °C (determined by DSC, second heating run after rapid cooling, 20 °C·min^–1^), and *T*_m_ = 48.8, 76.5, and 89.4 °C (determined by DSC, first heating run after rapid cooling, 20 °C·min^–1^) are commercial products purchased from NaturePlast (France) and used for the cast film preparations. Chloroform with purity >99.0–99.4% from Merck Company (Germany) was used as received.

### Pressed Film Preparation

Thin films for pressing were prepared by solution casting. PBSA was dissolved in chloroform to obtain a concentrated solution (10 wt%), which was then used to cast the film on a Teflon disc. The obtained PBSA thin films were cut into small circle-shaped pieces with a mean diameter of 14 mm, a thickness of 0.28 ± 0.03 mm, and an average mass *m* = 0.10 ± 0.05 mg using a cork borer. Pressed PBSA films, with a mean thickness of 0.13 ± 0.05 mm, were prepared from circle-shaped pieces of thin PBSA films on a hydraulic press with a force of pressure of the press jaws, 2, 2.5, and 5 t (metric ton) at a temperature of the press heating plate: room temperature (RT), 40, 60, 80, 90, and 100 °C for 1 to 5 min. The samples were then cooled in air to RT. The names of the specimens along with the processing conditions are given in Table 1.

**Table 1:** Thermal Properties from DSC and Phase Content from XRD of Commercial PBSA Pellet, Cast PBSA Thin Film, and Pressed PBSA Films with Names and Processing Conditions of the Analyzed Specimens^a^

	processing conditions			thermal properties							phase content	
specimens	*T* [°C]	*p* [t]	*t* [min]	*T*_g_ [°C]	*T*_m1_ [°C]	*T*_m2_ [°C]	*T*_m3_ [°C]	Δ*H*_m_ [J·g^–1^]	*X*_c_ [%]	texture	crystalline [%]	mesophase [%]
commercial PBSA pellet	−	−	−	–43.4	48.8	76.5	89.4	52.38	nd	−	nd	−
cast PBSA thin film	−	−	−	–44.1	45.3	65.5	92.1	65.69	51	S	51	−
pressed PBSA1	60	p.j.	1	nd	41.6	74.9	88.8	71.54	40	S	40	−
pressed PBSA2	60	p.j.	2	nd	41.3	74.7	88.7	71.89	39	S	39	−
pressed PBSA3	60	2	1	–45.0	44.8	−	96.1	60.17	26	CC	13.5	12.5
pressed PBSA4	60	2	2	nd	40.3	63.6	93.1	57.63	22	CC	11.5	10.5
pressed PBSA5	60	5	1	nd	42.1	77.2	92.9	65.85	25	CC	13.0	12.0
pressed PBSA6	60	5	2	–44.4	43.3	71.8	93.0	33.27	23	CC	11.8	11.2
pressed PBSA7	80	p.j.	1	nd	42.0	84.6	88.3	30.16	49	S	49	−
pressed PBSA16	90	2.5	2	–45.5	40.8	70.8	84.6	62.54	57	S	57	−

a *T*, pressing temperature; *p*, pressure; *t*, pressing time; *T*_g_, glass-transition temperature; *T*_m1–3_, melting temperatures; Δ*H*_m_, melting enthalpy; p.j., the pressure of the press jaws; nd, not determined; *X*_c_, relative crystallinity from XRD; CC, the film with the condis crystal-like mesophase; S, the film with spherulitic texture of the crystalline phase.

### Characterization Methods

#### Polarized Optical Microscope

All of the pressed films were observed with a POM Zeiss (Opton-Axioplan), equipped with a Nikon Coolpix 4500 color digital camera and a Mettler FP82 hot plate with a Mettler FP80 temperature controller. The specimens were placed on a microscope slide with a coverslip, and then, for specimens with a birefringence, the slide was heated and cooled while observing the phase changes.

#### Differential Scanning Calorimetry

Thermal characteristic of the pressed films was obtained using a TA-DSC Q2000 apparatus (TA Instruments, Newcastle, DE). The instrument was calibrated with a high-purity indium. DSC studies were carried out at a temperature from −60 to 120 °C with a rate of 20 °C·min^–1^ (I-heating run). All of the experiments were performed under a nitrogen atmosphere with a nitrogen flow rate of 50 mL·min^–1^, using aluminum standard sample pans. The *T*_m_ was taken as the peak temperature maximum of that melting endotherm (melting enthalpy, Δ*H*_m_), and *T*_g_ was taken as the midpoint of the heat capacity change of the specimen.

#### X-ray Diffraction

XRD studies were performed using a D8 Advance diffractometer (Bruker AXS, Karlsruhe, Germany) with a Cu–Kα cathode (λ = 1.54 Å) operating at 40 mA current and 40 kV voltage. The collection of data was performed by an LYNXEYE XE-T linear detector. The scan rate was 2.4°·min^–1^, with a scanning step of 0.02° in the range of 5 to 50° 2*θ*.

## Results and Discussion

The slow plastic deformation, where the polymer is compressed in the temperature range between *T*_g_ and *T*_m_, promotes the formation of order, as the chains are straightened and oriented during deformation. At high pressure and temperature, the chains become more mobile, which in turn leads to better order in the macromolecules and thus may introduce different degrees of order by crystallization or mesophase formation upon cooling.^18^ By doing so, it is possible to obtain different types of mesophase depending on the structure of the molecule. The idea is to apply high pressure/temperature to the polymer by heating it above its *T*_g_ but below its *T*_m_ and reduce the temperature. The crystals formed during the temperature change are then called the thermotropic mesophase, while the lyotropic mesophase is formed during concentration changes. The main factor determining the formation of the mesophase in the thermotropic case is a gradual temperature change and this has a great influence on the order of the different phases that occur one after the other.^27^ PBSA is known for its amorphous or semicrystalline structure, which may have an influence on specific *T*_g_ and *T*_m_. This depends on factors such as the composition of the copolymer, processing conditions, the ratio of butylene succinate to butylene adipate units in the copolymer, and any additives.

**Figure 1:**
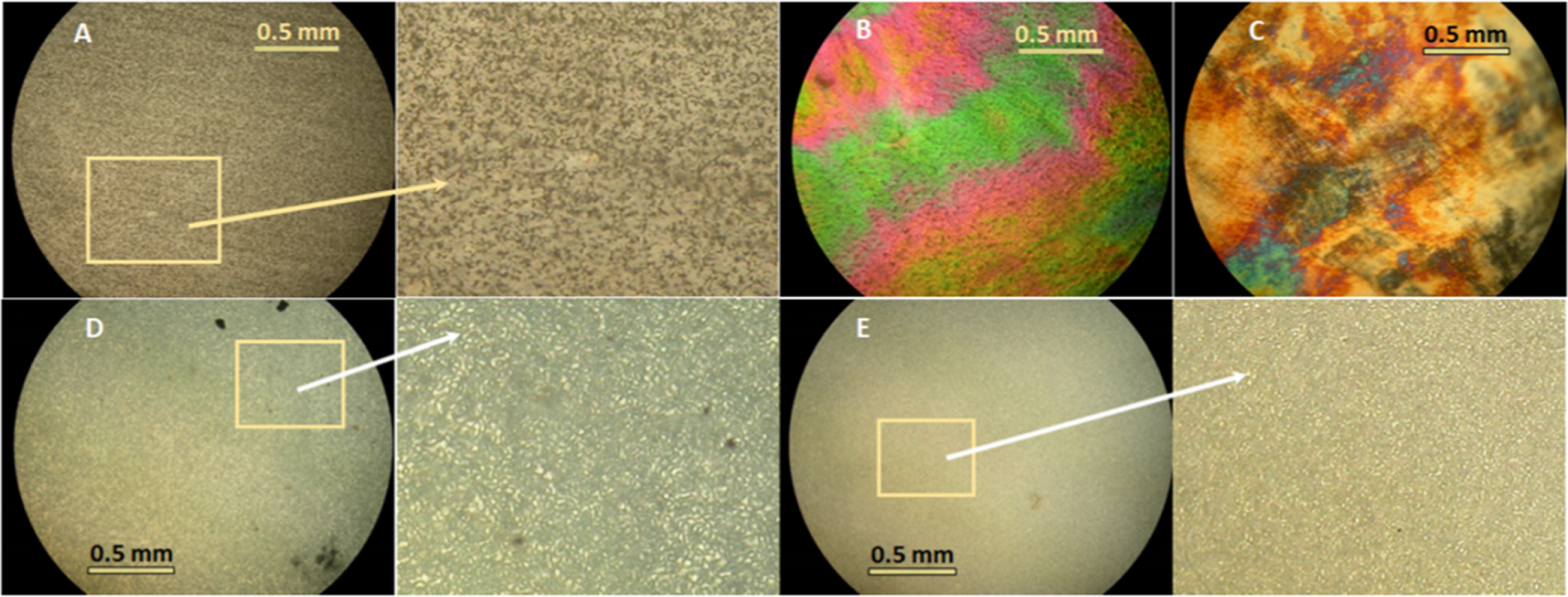
Representative POM photomicrographs of the surface of the cast PBSA thin film (semicrystalline material, (A)); pressed PBSA films obtained at a temperature of 60 °C and at a pressure of 2 t for 1 min (B) as well as at a pressure of 5 t for 2 min (C) (both color condis crystal-like mesophase); pressed PBSA films obtained at a temperature of 90 °C and at a pressure of the press jaws for 1 min (D) and at a pressure of 2.5 t for 2 min (E) (both typical spherulitic texture of the crystalline phase) (crossed polarizers, 25 °C, 100×).

The cast PBSA thin film before pressing is found to be a semicrystalline material based on the absence of birefringence under POM, thermal properties by DSC, and the presence of crystalline reflections by XRD. Orientational order does not occur naturally in polymer films. The condis crystal-like mesophase was obtained for PBSA under the influence of temperature and mechanical stress caused by pressing. Cast PBSA thin films were heated from RT to the temperature below the *T*_m_ = 92.1 °C and subsequently cooled to RT (i.e., above its *T*_g_ = −43.4 °C). Several different temperatures of RT, 40, 60, 80, 90, and 100 °C were selected due to the experience from previous results, indicating that the mesophase usually takes place slightly above the *T*_g_.^15,28^ For the experiment, the pressure of the press jaws, 2, 2.5, and 5 t, and the time from 1 to 5 min were also selected. It is assumed that when the specimen is cooled to RT, the polymer is expected to retain the structure of the condensed crystal mesophase. The phase behavior of obtained pressed films was investigated by observation of the optical texture using POM (Figure 1).

**Figure 2:**
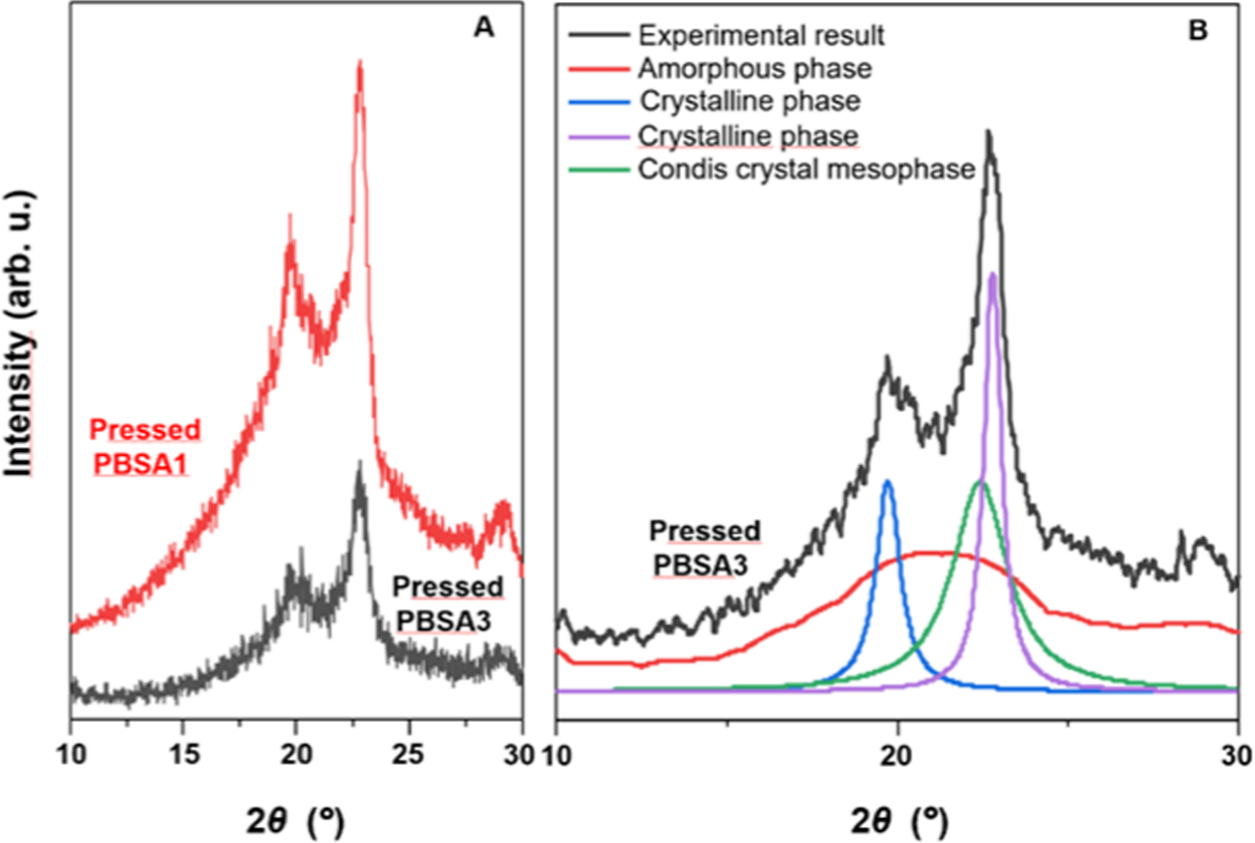
((A), left) Representative X-ray diffractograms of the pressed PBSA film obtained at a pressure of 2 t (color condis crystal-like mesophase, pressed PBSA3) and pressed PBSA film obtained at a pressure of the press jaws (typical spherulitic texture of crystalline phase, pressed PBSA1) for 1 min and at 60 °C; ((B), right) XRD diffraction patterns of PBSA3 and the corresponding peak deconvolutions. Scans were normalized to the maximal intensity of the most intense PBSA peak (2*θ* = 22.73°).

The X-ray diffraction analysis of pressed PBSA films confirmed the presence of a mesophase. This mesophase is characterized by the presence of crystalline reflections, indicating the existence of a condis crystal-like mesophase. Representative XRD patterns of pressed PBSA films obtained for 1 min and at 60 °C at a pressure of 2 t (PBSA3) and press jaws (PBSA1) are presented in Figure 2A, while in Figure 2B, the deconvolution of peaks for PBSA3 specimen is presented.

The pressed PBSA films reveal that the well-pronounced crystalline structure was detected, visible as sharp and intense peaks. In the case of films containing condis crystal-like mesophase, the detected peaks are significantly less intense, indicating a lower crystallinity of the system. Moreover, based on the peak deconvolution presented in Figure 2B, it can be concluded that the detected condis crystal-like mesophase was observed.

In the case of films containing a condis crystal-like mesophase, a significant decrease in crystallinity is observed. The relative crystallinity of pressed PBSA films with a spherulitic texture, calculated using the peak decomposition method, ranges from 39% for the pressed PBSA films obtained only under jaw pressure to 57% for the pressed PBSA films obtained at 90 °C, while that of the pressed PBSA film with a condis crystal-like mesophase ranges from 22 to 26% (see Table 1). The condis crystal-like mesophase content of the pressed PBSA films ranged from 10.5 to 12.5%; therefore, one can notice that the mesophase and crystal phases are formed in nearly equal proportions.

**Figure 3:**
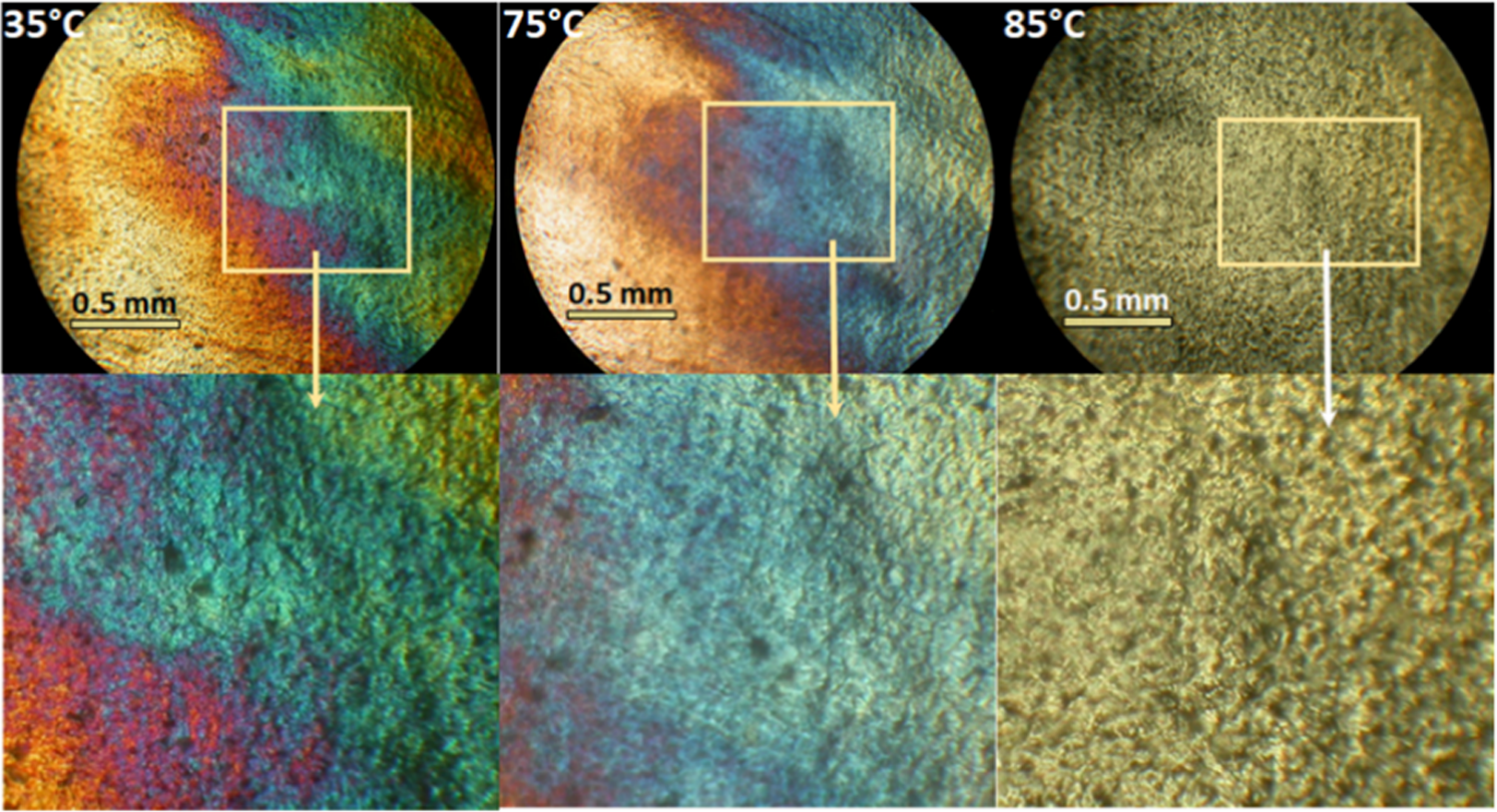
Photomicrographs of the pressed PBSA film with the condis crystal-like mesophase during heating at 35, 75, and 85 °C (crossed polarizers, 100×).

In parallel with the XRD, birefringence analysis was used (under POM) to confirm the long-range structural orientation in the mesomorphic pressed PBSA films obtained at different pressing temperatures. Birefringence occurs in a polymer when the molecular chains within the polymer align parallel to each other under stress. This alignment causes the polymer to have different refractive indices along different directions, resulting in the splitting of light into two polarized components.^29^ The thermal stability of the condis crystal orientation was evaluated by heating the polymer films under a POM equipped with a hot plate. The photomicrographs show a typical example of birefringence (Figure 3).

Figure 3 illustrates the pressed PBSA film with the condis crystal-like mesophase under crossed polarizers at 35 and 75 °C. By increasing the temperature to 85 °C, the condis crystal-like mesophase lost its birefringence and was transformed into a crystalline phase. The formation of a condis crystal-like mesophase and an increased Δ*H*_m_ is a precursor to the crystalline state.^1^

In contrast, films pressed at temperatures from 80 to 100 °C showed only a crystalline phase characterized by the presence of reflections at wide angles in the XRD scattering method (see Figure 2A) and the absence of birefringence under POM (see Figure 1A,D,E), indicating the dominant presence of the crystalline fraction.

Mesophase is an intermediate phase between the liquid and solid states that exhibit partial ordering and mobility. In a mesophase, large-amplitude motions, such as translation, rotation, and conformational motion, are not fully frozen in the ordered phase. The variations in chain packing and mobility of coupled amorphous chain portions in polymers impact their mechanical properties, including both initial resistance to tensile strain and large strain behavior. More specifically, stable crystalline forms exhibit higher Young’s modulus and can withstand lower deformation under mechanical stress compared to condis mesophases, which have a more disordered structure. The transition from condis to crystal mesophase occurs when the chains have sufficient mobility after energy is supplied to allow the chain rearrangements’ conformation and packing into the unit cell. The transformation of a metastable structure (molecular packing in unit condis cell is looser and more disordered) into a stable (energetically favorable) structure involves the contraction of the unit cell, which results in a more ordered and thermodynamically stable crystal structure and affects the properties of the materials. Condis mesophase of the polymer is metastable and undergoes a spontaneous transformation into a more stable crystalline structure when annealed above a critical temperature (in our case 85 °C), which allows for rearrangements of chain conformations and results in a higher coupling of amorphous and crystalline chain segments, affecting their stiffening compared to conformationally disordered arrangements. This transformation leads to differences in chain packing and mobility, affecting mechanical properties, such as the varied morphology of polymers, with the degree of order and packing of molecules within the crystal unit cell playing a key role.^30^ For example, condis crystal-like mesophases in polymer blends offer a significant advantage in drug delivery applications due to their enhanced stability and faster dissolution properties compared to amorphous and pure crystalline phases.^31^ Polymers having a condis crystal-like mesophase are also effective modifiers during polymer blending, which allows for more efficient processing and a material with improved physicomechanical properties.^32^ What this means is that due to the existence of the mesophase, (bio)degradable polymers can be used more widely. This is due to their improved properties that are adapted to wider applications, eliminating conventional polymers, which are also more sustainable.

**Figure 4:**
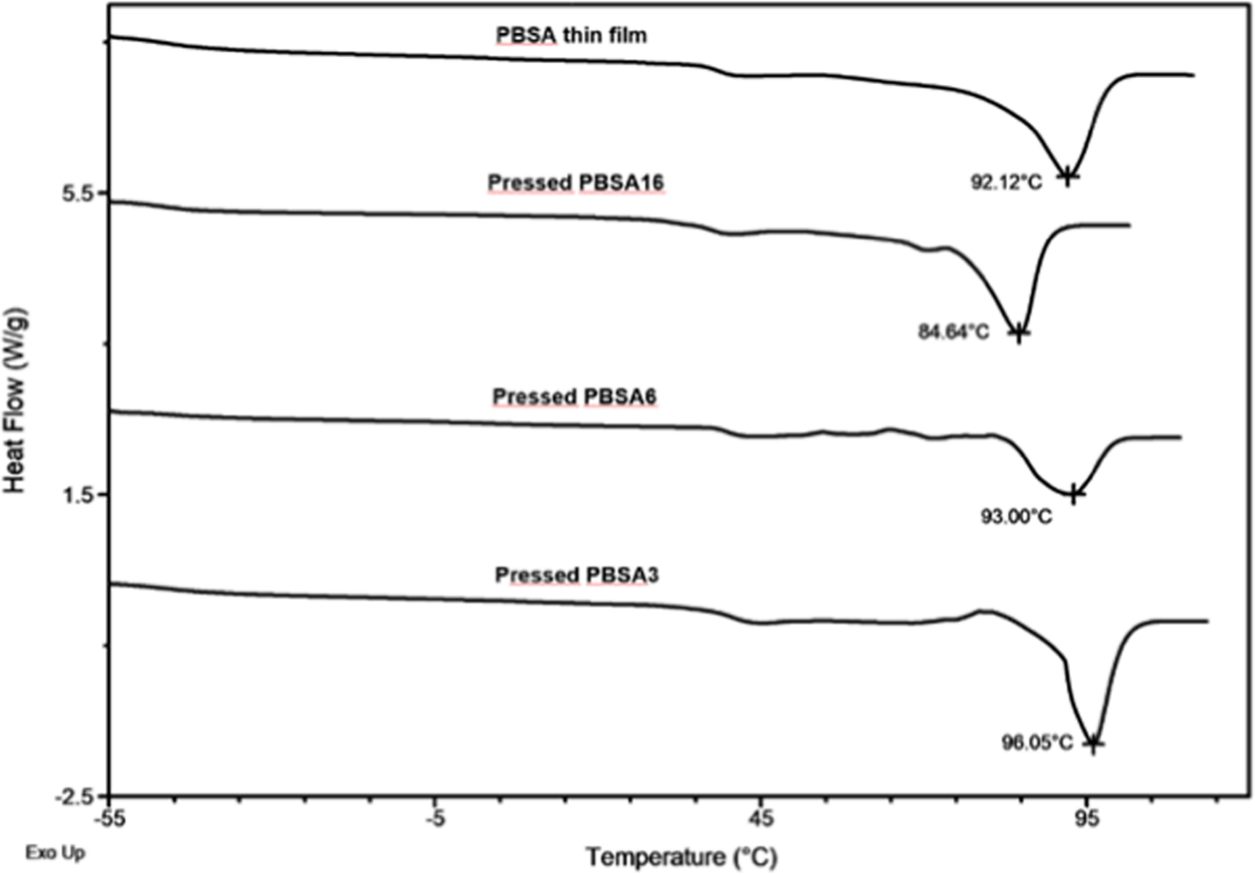
Representative DSC curves of the cast PBSA thin film before processing (semicrystalline material) and pressed PBSA films processed above the *T*_g_ of PBSA: 60 °C (with a pressure of 2 t for 1 min, pressed PBSA3 as well as of 5 t for 2 min, pressed PBSA6), both color condis crystal-like mesophase and 90 °C (with a pressure of 2.5 t for 2 min, typical spherulitic texture of the crystalline phase, pressed PBSA16).

DSC analysis (Table 1 and Figure 4) confirmed the presence of a condis crystal-like mesophase, as indicated by the high Δ*H*_m_ value (for the nematic mesophase, Δ*H*_m_ is low^15^).

Copolymers containing poly(butylene adipate) (PBA) do not undergo cold crystallization and exhibit bimodal melting due to their polymorphic nature upon crystallization. The β-phase is the dominant polymorphic form, but upon heating (in our case during processing), there is a transition from the less stable β-phase to the more stable α-phase.^33^ Therefore, PBSA shows multiple melting points.

## Conclusions

In this study, experiments with PBSA films have demonstrated that films with different orientational orders of the crystal phase can be prepared under the influence of temperature and mechanical stress. In one case, in particular, the films treated under mechanical pressure and at a time above the *T*_g_ of PBSA at 60 °C, a condis crystal-like mesophase was obtained, which was characterized by birefringence under POM and the presence of reflections at wide angles under the XRD scattering method. This phenomenon was observed when the PBSA films were heated from room temperature to a value below *T*_m_ = 92.1 °C and then allowed to cool to RT on its own. As the PBSA films are cooled to RT, the polymer retains the condis crystal mesophase structure. The results also showed that films pressed at higher temperatures (from 80 to 100 °C) exhibited only a crystalline phase characterized by the presence of wide-angle reflections in the XRD scattering method (data in Figure 2A). Likewise, an absence of birefringence was also noted in the POM examination of these films.

Chain entanglements occurring in the amorphous phase of semicrystalline polymers influence many of the macroscopic material properties. Understanding and controlling these entanglements is essential for optimizing the properties and performance of polymer-based products in various sectors. The formation of a condis crystal-like mesophase with high chain mobility, allowing long-range chain diffusion, facilitates chain disentanglement.^34^ In the condis mesophase, rapid reptation (movement of entangled polymer chains in a characteristic way resembling snakes crawl over each other) results in the elongation of folded chains in such crystals. These driving forces can trigger significant structural changes in such a mesophase, particularly when it comes to reducing the surface free energy of the folded chain lamella or mechanical energy during drawing. The formation of extended chain crystals allows for the creation of polymer materials with tailored properties. This complex interplay between molecular dynamics and structural changes within polymer materials under specific conditions determines their applications.^35^ This phenomenon highlights the importance of understanding structure-property relationships in materials science and engineering and the need for further research.
